# iKNOW—Supporting the counseling of women with hereditary risk of breast and ovarian cancer with digital technologies: A randomized controlled trial

**DOI:** 10.1016/j.gimo.2024.101892

**Published:** 2024-09-06

**Authors:** Markus A. Feufel, Dorothee Speiser, Stephen Schüürhuis, Konrad Neumann, Marie Keinert, Steffi Stegen, Gudrun Rauwolf, Maren Heibges, Viola Westfal, Laura Besch, Christine Olbrich, Katharina Klein, Isabell Witzel, Friederike Kendel

**Affiliations:** 1 Division of Ergonomics, Department of Psychology and Ergonomics, Technische Universität Berlin, Berlin, Germany; 2 Department of Gynecology with Breast Center, Hereditary Breast and Ovarian Cancer Center, Charité—Universitätsmedizin Berlin, Berlin, Germany; 3 Institute of Biometry and Clinical Epidemiology, Charité—Universitätsmedizin Berlin, Berlin, Germany; 4 Department of Clinical Psychology and Psychotherapy, Friedrich-Alexander-Universität Erlangen-Nürnberg, Erlangen-Nuernberg, Germany; 5 BRCA-Netzwerk e.V., Hilfe bei familiären Krebserkrankungen, Bonn, Germany; 6 Institute of Vocational Education and Work Studies, Education for Sustainable Nutrition and Food Science, Technische Universität Berlin, Berlin, Germany; 7 Institute of Sexology and Sexual Medicine, Charité – Universitaetsmedizin Berlin, Berlin, Germany; 8 Gender in Medicine, Charité—Universitätsmedizin Berlin, Germany Corporate Member of Freie Universität Berlin, Humboldt—Universität zu Berlin, and Berlin Institute of Health, Berlin, Germany; 9 Department of Gynecology, University of Zurich, Switzerland

**Keywords:** Digital health, Genetic counseling, Gynecology, Hereditary cancer, Risk communication

## Abstract

**Purpose:**

We developed the online-based counseling tool iKNOW for women with a pathogenic germline variant in *BRCA1/2* to improve risk understanding and quality of life.

**Methods:**

With a randomized controlled trial, we investigated the efficacy of iKNOW with regard to risk understanding (primary endpoint), quality of life, risk perception, and anxiety (secondary endpoints). Self-report questionnaires were administered to *N* = 217 women with a pathogenic variant in *BRCA1/2* before counseling (T0), immediately after (T1), 4 weeks after (T2), and 6 months after (T3).

**Result:**

Deviations between self-assessed and calculated cancer risks tended to be smaller in the intervention group than in the control group but no longer significantly after adjustment for multiple testing. In the intervention group, the proportion of women with a correct understanding of breast cancer risk at T3 was higher (30.7% vs 14.7%; *P* = .032). There were no differences in secondary endpoints.

**Conclusion:**

iKNOW tends to positively influence the understanding of familial cancer risk. At the same time, it does not negatively influence any of the secondary endpoints. However, converging evidence suggests that iKNOW seems to affect the quality of counseling processes and can thus be used as a paradigm for reinventing the notion of efficient, digital care.

## Introduction

Digital technologies offer new opportunities to personalize, update, and standardize health information. By automatically tailoring information to patient needs, they have the potential to improve medical counseling, particularly in complex settings, such as personalized and individualized medical care. An excellent example of complex requirements for counseling is hereditary breast and ovarian cancer (HBOC): women with a pathogenic germline variant in BRCA (gBRCA) have a significantly increased risk of developing breast and ovarian cancer (OC) compared with the general population,^1^ with a cumulative breast cancer risk to age 80 of 72% for *BRCA1* and 69% for *BRCA2*, and an OC risk of 44% and 17%, respectively.^2^ When counseling these women, various measures are discussed to counteract the increased risk. Risk management includes above all intensified surveillance programs for early breast cancer detection or prophylactic surgery, which must be weighed against the background of personal risk, family history, life circumstances, and preferences.^3^

A prerequisite for the discussion of possible options to individually manage the risk is the communication of cancer risks across the life span and for different time periods. According to the guideline of the German Consortium HBOC (GC-HBOC), these risks are calculated individually for each counselee using the validated Breast and Ovarian Analysis of Disease Incidence and Carrier Estimation Algorithm (BOADICEA) algorithm.^4^ The consulting physician must then translate the calculated risks into easy-to-understand risk formats. An understanding of the 10-year risk is crucial because the calculated risk over this period will determine the next steps to be taken.

Women who seek specialized counseling have to absorb a great amount of new and complex information, and many women may feel overwhelmed by the information they receive. In a previous study, we found that the majority of counselees were unable to correctly recall their risks after counseling.^5^ Another challenge in counseling women with BRCA is dealing with the emotional impact of the diagnosis. Learning that they carry a pathogenic variant, which significantly increases their risk of cancer, can cause great distress, evoking feelings of anxiety and uncertainty, which, in turn, can make it difficult to understand medical information.

To date, there are only a few digital tools available for women with a pathogenic germline variant in *BRCA1/2*, and most of them focus on decision support and are either designed for patient or physician use.^6^, ^7^, ^8^, ^9^, ^10^ The Australian “iPrevent” tool,^11^ for instance, depicts individualized risks and allows physicians or women to display and compare risk reductions associated with preventive options. An exploratory study demonstrated good usability and acceptability of iPrevent.^12^ So far, detailed clinical evaluation results are lacking for most of the existing digital tools, and the majority only support part of the decision-making process (eg, risk communication, preference elicitation, or easy-to-comprehend explanations of familial cancer risks and the preventative options provided). In the context of this research project, we developed the online counseling tool iKNOW. To our knowledge, this is the first digital tool to be used during (and after) counseling to support the doctor-patient interaction. iKNOW helps physicians to tailor information contents to the specific needs of the counselee (eg, based on their pathogenic germline variant related to *BRCA1* or *BRCA2* and whether and what kind of therapy they have already started) and relate individualized risk estimates with explanations of the preventive options. If required, counselees can reexamine these options using iKNOW after counseling.

The iKNOW tool has been developed against the background of a growing global shortage of doctors on the one hand and a rapid growth of specialized genetic knowledge on the other.^13^ At the same time, patients have high expectations of the quality of their doctor-patient communication.^14^ To support doctors and their patients in disseminating and discussing expert knowledge, we have developed iKNOW around 5 empirically based principles, which may help researchers from other countries to develop locally adapted versions of the counseling tool.^15^ Specifically, iKNOW was designed to (1) implement the current standard of care, (2) support personalization and standardization of the counseling process, (3) make complex information easily accessible, (4) respect the privacy rights of counselees, and (5) use systematic and iterative evaluation with users in mind. To facilitate physician risk communication and patient risk understanding, we referred to the research of Gigerenzer et al^16^ on principles of transparent risk communication based on a combination of numerical and visual formats for representing absolute risk estimates.

With iKNOW, we aimed to improve counseling for women with a pathogenic germline *BRCA1/2* variant. We hypothesized that the counseling tool would lead to a more accurate understanding of 10-year breast cancer and OC risk at 6 months after the first counseling (primary outcome). We also hypothesized that women in the IG would report lower use of health care services, lower risk perception, lower cancer worry, lower state anxiety, and higher quality of life (secondary outcomes). In addition, we compared satisfaction with counseling (range from 0 = very unsatisfied to 12 = very satisfied) and evaluation of counseling contents (range from 0 = very bad to 18 = very good) in both groups.

## Materials and Methods

### Participants

We recruited women attending a large HBOC Center from December 2019 until March 2021. Women were eligible to participate if they were carrying a pathogenic germline variant in *BRCA1/2*, were between 18 and 70 years of age, and had internet access. Reasons for exclusion were previous numerical risk consultation for familial breast or OC, severe psychiatric comorbidity, and insufficient knowledge of the German language. All participants were informed about the study and provided written informed consent before enrollment. All participants were from different families with pathogenic germline variants in *BRCA1/2*. The study was approved by the ethics commission of the institution (EA4_217_17) and was registered in the German Register of Clinical Trials (ID: DRKS00020126).

### Design and procedure

The study was conducted as a nonblinded, monocenter, randomized controlled intervention trial with 2 parallel groups according to consolidated standards of reporting trials guidelines. Consultation, study management, and data analyses were carried out independently of each other. Participants were randomly assigned to consultation using the online-based counseling tool iKNOW (intervention group, [IG]) or standard consultation (control group, [CG]) in a 1:1 ratio using block randomization (block size 8, R Version 3.6.1). Sequentially numbered, opaque, and sealed envelopes contained the predetermined assignments and were sequentially opened after the respective participant’s name had been written on the envelope.

Counseling during the study was performed independently of the genetic analysis and the communication of the genetic test results to give counselees time to decide whether to undergo individualized counseling. Specifically, 51% of the counselees presented for counseling in the same year they had received the genetic test result, and 49% had received it more than 1 year before deciding on a second counseling appointment. In both the IG and CG, counseling followed a nondirective standard procedure according to the guidelines of the centers of the GC-HBOC and lasted 60 to 90 minutes each. In the IG, counseling was provided using the online counseling tool iKNOW, and the women could continue to access the contents provided in the tool after the counseling.

The women were asked to complete self-report paper/pencil questionnaires immediately before (T0) and after (T1) counseling at the study center. T2 and T3 measurement points (4 weeks and 6 months after counseling, respectively) were mailed. Because of the COVID-19 pandemic, the consultation had to be changed to an online format, and, accordingly, data collection to online questionnaires.

If a questionnaire was not returned, up to 3 reminders were sent at T2 and T3. It was ensured that each woman received only 1 questionnaire format (paper/pencil vs online) throughout the survey period. As a priori blinding of physicians was not possible in this setting, potential experimenter bias was addressed by a common structured study protocol with training in both conditions.

**Figure 1:**
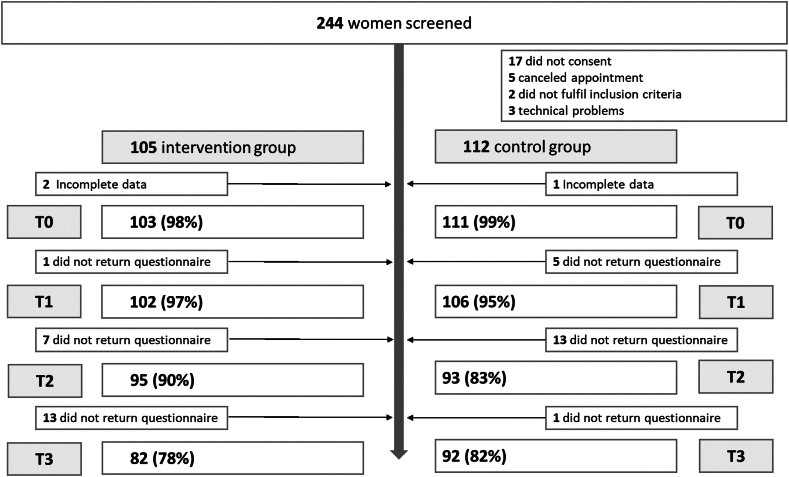
**Flowchart of participants**.

Initially, 244 women were screened, of whom 82 women in the IG and 92 women in the CG completed the questionnaires 6 months after baseline (T3). Figure 1 shows the reasons for dropout over time. Following changes during the COVID-19 pandemic, *N* = 103 women received counseling via video consultations, in which the online counseling tool was shared with the counselees via the video conference software.

### Intervention: Description of the online-based counseling tool iKNOW

The online counseling tool iKNOW consists of 4 content areas: “Pedigree and risk presentation,” “Genetics and family,” “Screening and prevention,” and “Support and lifestyle.” The content of the tool is evidence based and easy to update in the face of rapidly changing data. It also complies with data protection and privacy guidelines. At the beginning of each consultation, the tool can be used to adapt the counseling contents to the counselees’ individual situation by filtering for 4 variables: (1) type of pathogenic germline variant (BRCA1 or BRCA2), (2) cancer diagnosis (no cancer/breast cancer unilateral/breast cancer bilateral/OC/metastasized disease), (3) ongoing systemic therapy (yes/no), (4) prophylactic surgery (no prophylactic surgery/salpingoophorectomy/mastectomy).

For each woman, individual, age-related numerical risks for breast and/or OC based on the BOADICEA algorithm are presented in the tool. The risk is presented using icon arrays and curve plots, allowing for direct comparison of the individual risk with that of the general population over time for breast and OC separately.

Whereas the physician’s version of the tool contains mainly graphics for explanation during the counseling appointment, the counselee’s version has additional explanatory text in a simple and nontechnical language. Short summaries highlight key points. The evidence-based approach is emphasized by providing references. At the end of each counseling session, the content is made available to the counselee upon request. That is, the tool allows the physician to disclose the content that has been explicitly requested and conceal all remaining contents the patient wishes not to see, which is in line with the “right to not know” under the German Genetic Diagnostics Act. For more detail, see Speiser et al.^15^

### Main outcome measures

Based on a preliminary study, risk understanding after 6 months was defined as the primary endpoint.^5^^,^^15^ Understanding of numerical 10-year risk was assessed with a single item (adapted from Gigerenzer, Mata, and Frank^17^). Participants were asked to “imagine 100 women in exactly the same situation” as themselves (in terms of age, pathogenic germline variant and family history) and to estimate how many of these women would develop breast or OC in the next 10 years. Subjective risk perception was assessed with an item similar to risk perception (“How high do you think your own risk is of developing breast or ovarian cancer [again] within the next 10 years?”) using verbal labels ranging from 0 “extremely low” to 10 “extremely high.”

To assess cancer worry, we used an abbreviated version of the Cancer Worry Scale^18^ consisting of 4 items. State anxiety was assessed using the 5-item subscale of the State-Trait Anxiety Inventory.^19^ Overall health in the past 4 weeks was measured with the single item rated on a 5-point Likert scale (ranging from 1 = “poor” to 5 = “excellent”).^20^ Clinical data were provided by physicians at baseline.

Satisfaction with the consultation was assessed using an adapted and shortened version of the patient satisfaction questionnaire (ZUF-8) with 4 items.^21^ Seven self-constructed items were used to measure the quality of counseling content. The tool’s usability was measured using the subscales usefulness, usability, and visual esthetics of the Modular Evaluation of Key Components of User Experience (meCUE) questionnaire.^22^

### Statistical analysis

The statistical analysis was performed with the available data set, comprising all study participants who completed the study as randomized and had available outcome measures at T3. Percentages of missing values in the primary outcome measures are displayed in Supplemental Table 2. Given the assumption that women with poor risk comprehension are more likely to refrain from providing a risk assessment, we suspect that missing data are not at random. Therefore, we opted against conducting a multiple imputation analysis, recognizing the potential for bias in our estimates.

The sample size planning was founded on the expectation of a reduction of over 57.9% in the absolute deviation of self-assessed and calculated cancer risks attributed to the intervention. This corresponds to a shift effect of λ = 0.865 on the logarithmic scale, which is associated with a relative treatment effect of P(X < Y) = 0.681. To estimate the relative treatment effect from λ, we used data from a pilot study.^5^ With a sample size of *n* = 80 per group, the Mann-Whitney U test has a power of over 80% at α = 0.05 (nQuery Advisor 7.0). Considering a 20% dropout rate, a total of at least *N* = 200 participants (*n* = 100 per group) had to be recruited for the study to ensure sufficient power at the assumed effect size.

All quantitative variables were summarized using the mean and standard deviation, whereas categorical variables were assessed through absolute and relative frequencies. Accurate risk understanding was defined as the absolute difference between the computed 10-year breast and OC risk (determined by the BOADICEA algorithm) and the numerical risk estimates provided by the counselees. To facilitate interpretation, these absolute deviations were dichotomized: An absolute deviation (under- or overestimation) of less than 5% was considered a correct estimation, whereas a deviation of more than 5% was considered as incorrect. We used the nonparametric Mann-Whitney-U test to compare absolute differences between IG and CG in risk understanding at T3. The evaluation of the binary variable was carried out with Pearson’s χ^2^ test.

Analyses adjusting for baseline were performed with multiple regression analysis. For the absolute risk deviation, we used a linear regression model with study group and baseline as independent variables. The primary outcomes underwent a Box-Cox transformation to meet the assumptions of normality (Supplemental Remark 1). In a similar fashion, logistic regression with baseline adjustment was used to assess the impact of the intervention on the dichotomized variable. To perform pairwise comparisons of assessments at T0, T1, T2, and T3 within the respective groups, we utilized the nonparametric Brunner-Munzel test for paired data.

For the ordinal-scaled variable “subjective quality of life,” we provided frequency distributions for the time points T0, T2, and T3. We used random slope linear models with baseline and group as fixed effects to analyze the outcomes “subjective risk perception,” “cancer worry,” and “state anxiety.” For the analysis of “use of medical services,” a sum score was generated to illustrate the extent of additional doctor visits, and a *t* test was applied to examine whether there were differences between the IG and the CG at the respective measurement time points. The variables “satisfaction with counseling” (assessed on a scale ranging from 0 = very unsatisfied to 12 = very satisfied) and “evaluation of counseling content” (rated on a scale from 0 = very bad to 18 = very good) were compared between the IG and the CG at each respective time point through *t* tests.

In the primary analyses, we conducted a comparison of absolute deviation in risk estimates for both breast and OC. To address the issue of multiple testing, we present both Bonferroni-adjusted and unadjusted *P* values. In the secondary analyses, it is important to note that all *P* values were considered part of exploratory data analysis. All analyses with *P* values below a 2-tailed α level of 0.05 were considered statistically significant. The analyses were performed with IBM SPSS Statistics version 27 or R version 4.1.2.

## Results

The average age of the women who participated was 40.4 years, and the majority had a partner, higher education, were employed, and had children. About two-thirds of the women had a pathogenic variant in *BRCA1* and one-third had a pathogenic variant in *BRCA2*. Almost half of the women had a previous cancer diagnosis (86.1% breast cancer and 15.8% OC). Almost all women (93.5%) had a family history of (breast/ovarian) cancer and 23.9% had already undergone prophylactic surgery (Table 1).

**Table 1:** Sample characteristics at baseline (T0) according to treatment condition (IG, CG)

	Total (*N* = 217)	IG (*N* = 105)	CG (*N* = 112)
**Sociodemographic Characteristics**			
Age, y, M (SD)	40.4 (10.8)	39.4 (10.8)	41.3 (10.8)
With partner, *n* (%)	151 (70.2)	71 (67.6)	80 (72.7)
Higher education,^a^*n* (%)	160 (74.1)	74 (70.5)	86 (77.5)
Employed, *n* (%)	134 (61.8)	66 (62.9)	68 (60.7)
Biological children, *n* (%)	126 (59.2)	63 (61.2)	63 (57.3)
**Type of the pathogenic variant**			
BRCA1, *n* (%)	138 (63.6)	69 (65.7)	69 (61.6)
BRCA2, *n* (%)	79 (36.4)	36 (34.3)	43 (38.4)
**Additional clinical variables**			
Diagnosis of cancer, *n* (%)	101 (47.2)	49 (47.1)	52 (47.3)
Breast cancer, *n* (% of patients)	88 (87.1)	43 (87.8)	45 (86.5)
Ovarian cancer, *n* (% of patients)	16 (15.8)	6 (12.2)	10 (19.2)
Other, *n* (% of patients)	2 (2.0)	2 (4.1)	0 (0.0)
Missing, *n*	3	1	2
Familial cancer, *n* (%)	202 (93.5)	96 (92.3)	106 (94.6)
Missing, *n*	1	1	0
Prophylactic surgery	51 (23.9)	20 (19.4)	31 (28.2)
Mastectomy, *n* (% of patients)	35 (68.6)	13 (65.0)	22 (71.0)
Unilateral, *n* (% of patients)	6 (17.1)	3 (23.1)	3 (13.6)
Bilateral, *n* (% of patients)	29 (82.9)	10 (76.9)	19 (86.4)
Oophorectomy, *n* (% of patients)	28 (54.9)	8 (40.0)	20 (64.5)
Salpingectomy, *n* (% of patients)	8 (15.7)	1 (5.0)	7 (22.6)
Missing, *n*	4	2	2

*CG*, control group; *IG*, intervention group; *M*, arithmetic mean; *SD*, standard deviation.

a Defined as German “Abitur” (ie, final exam after 12 to 13 grades of schooling).

### Primary endpoint: Absolute deviations of patients’ risk estimates from calculated risks

**Figure 2:**
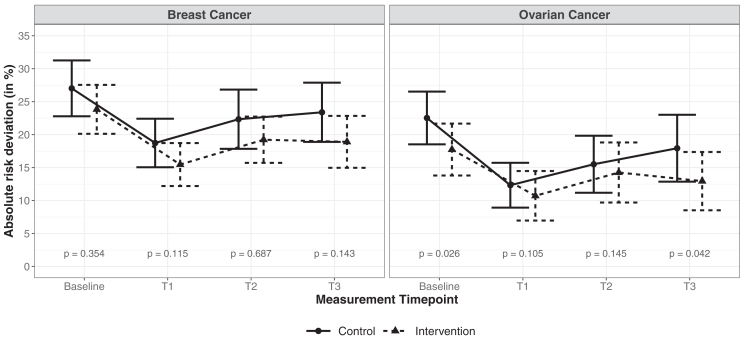
**Absolute deviations of patients' risk estimates from the statistically calculated risks for IG (dashed lines) and CG (solid lines).** Immediately after the consultation (T1), the difference between the patients' estimates and the calculated risk becomes smaller and then increases again. Unadjusted *P* values are calculated using the Mann-Whitney-U test. The error bars represent 95% confidence intervals for the estimated means.

Six months after baseline (T3), the mean absolute deviation of the patients’ estimates from the calculated breast cancer risk was 18.9% [14.9, 22.8] in the IG, and 23.4% [18.9, 27.9] in the CG (Mann-Whitney U test: P(CG > IG) = 0.57, 95% CI [0.48, 0.66], *P =* .143, $p_{adj}=0.286$), ie, absolute risk deviations tend to be larger in the CG at T3, but there was no statistically significant difference (Figure 2). To account for potential confounding by baseline differences, we fitted a linear regression model adjusting for baseline. The adjusted analysis yielded a similar result (group difference IG − CG on Box-Cox-transformed scale −0.74, 95% CI [−1.7, 0.17], *P =* .109; Box-Cox parameter: λ=0.34) (Supplemental Table 1A).

For OC, the corresponding deviations were 12.9% [8.5, 17.4] in the IG and 17.9% [12.9, 23.0] in the CG (Mann-Whitney U test: P(CG > IG) = 0.60, 95% CI [0.51, 0.70], *P =* .042, $p_{adj}=0.084$). However, after adjusting for multiple testing, the difference in absolute deviation was no longer statistically significant (Figure 2). After adjusting for baseline differences, the deviation from the actual risk was still smaller in the IG, but there was no statistically significant difference (group difference IG − CG on Box-Cox-transformed scale −0.64, 95% CI [−1.3, 0.05], *P =* .070; Box-Cox parameter: λ=0.14) (Supplemental Table 1A).

### Percentage of correct vs incorrect risk estimates

**Figure 3:**
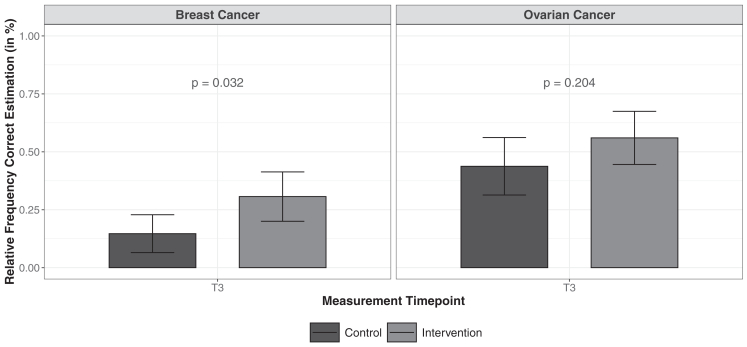
**Percentage of correct risk estimates for IG (light grey bars) and CG (dark grey bars) with a tolerance range of 5% based on the deviation of the estimates from the actual risk.***P* values are calculated using the χ2 test. The error bars represent 95% confidence intervals for the estimated proportions.

A dichotomized variable with a tolerance range of 5% was used to consider correct vs incorrect risk estimates. After 6 months, women in the IG had a higher percentage of correct risk estimates for breast cancer compared with women in the CG (χ^2^ test: 30.7% vs 14.7%; OR = 2.6, 95% CI [1.1, 5.8], *P =* .032) (Figure 3). Adjustment for baseline differences using logistic regression showed that the intervention was still associated with a significant increase in correct risk estimates as the odds for a correct estimation are 2.5 times higher in the IG (OR = 2.5, 95% CI [1.1, 5.8], *P =* .027) (Supplemental Table 1B).

The percentage of correct risk estimates of OC did not differ significantly between IG (56.0%) and CG (43.8%) (χ^2^ test: OR = 1.6, 95% CI [0.84, 3.2], *P* = .204) (Figure 3). Baseline adjustment using logistic regression did not lead to any qualitative changes in this finding (OR = 1.4, 95% CI [0.66, 2.8], *P =* .407) (Supplemental Table 1B).

### Post-hoc analyses to understand missing values

There were a considerable number of missing data points in the primary outcome “absolute risk deviation.” These missing values can be attributed to either the absence of subjective risk estimations (Supplemental Table 2) or the inability to calculate cancer risk for certain cases. For instance, it is not possible to calculate cancer risks for women with a diagnosis of OC and for those who underwent prophylactic surgery. Consequently, in our subsequent analysis of the missing data, we excluded all cases with missing values for calculated cancer risk.

Regarding the OC absolute risk deviation, we found that 18.5% and 17.9% of outcome values were missing for the IG and CG at T3, respectively, after excluding cases with missing calculated risk values. For breast cancer absolute risk deviation, the percentages of participants with missing values were 19.4% for the IG and 14.8% for the CG.

Comparing the group of women with and without missing values for OC at T3 revealed that women with missing values more often had a diagnosis of cancer (53.3% vs 37.4%), less often had a higher education (64.5% vs 79.0%), and less often were employed (48.4% vs 69.1%). Comparable patterns were apparent in relation to breast cancer (Supplemental Table 4). Because women with poor risk comprehension are more likely to not provide a risk assessment, it is likely that the missing data are not at random. Consequently, using multiple imputation could exacerbate bias in the results. Therefore, we have opted not to present the results of an analysis using multiple imputation.

### Differences in risk perception, anxiety, and quality of life

Linear models with random effects indicate that IG and CG did not differ in subjective risk perception (IG intercept 5.3, 95% CI [5.0, 5.6], CG intercept 5.1, 95% CI [4.8, 5.5], *P =* .454) (Supplemental Table 5), illness anxiety (IG intercept 10.1, 95% CI [9.7, 10.4], CG intercept 10.2, 95% CI [9.9, 10.6], *P =* .580) (Supplemental Table 6), and general anxiety (IG intercept 9.5, 95% CI [9.1, 9.9], CG intercept 9.4 95% CI [9.0, 9.7], *P =* .597) (Supplemental Table 7). IG and CG also did not differ notably in subjective quality of life at any time point (χ^2^ test, T0: *P =* .390, T2: *P =* .989, T3: *P =* .445) (Supplemental Table 8).

### Differences in the use of medical services and satisfaction with the counseling

After 6 months (T3), women from the CG did not, on average, use more medical services at the HBOC center or externally (*t* test: IG mean 0.96 (1.2) visits, CG mean 1.4 (2.1) visits, *P =* .070 and IG mean 1.4 (2.4) visits, CG mean 2.1 (3.8) visits, *P =* .176, respectively). More than one-third of respondents (34.5%) reported that they had not had any additional examinations. The proportion of women who did not have any additional medical services did not differ significantly between IG (35.5%) and CG (33.6%). There were also no notable differences between women in CG and IG in terms of counseling satisfaction (*t* test: IG mean 11.0 (1.4), CG mean 11.0 (1.8) at T1 *P =* .786; IG mean 9.6 (2.3), CG mean 9.4 (2.4) at T3 *P =* .741). However, women in the IG rated the content of the counseling more positively than women in the CG at all measurement points (*t* test: T1: IG mean 15.6 (2.3), CG mean 14.8 (2.7) *P* = .023; T2: IG mean 14.6 (3.2), CG mean 13.1 (3.7) *P =* .004*;* T3: IG mean 14.3 (3.5), CG mean 13.1 (3.7), *P =* .030). The usability of the counseling tool (evaluated only by women in the IG) was rated as “very satisfied” by 54 of 99 (54%) women at T1 and by 54 of 63 (85%) women at T3.

## Discussion

We developed and evaluated a counseling tool that can be used in the context of HBOC risk counseling by both doctor and patient during the appointment and by counselees at home afterward. To the best of our knowledge, there is currently no comparable application that provides digital evidence- and guideline-based information to physicians and patients during (and after) counseling in an individualized, standardized, simplified, and continuously updated form. In our results, we observed effects that go in the expected direction and are indicative of an improvement of the numerical understanding of the 10-year risks of breast and OC compared with standard counseling. However, these differences are no longer significant after Bonferroni correction. At the same time, we did not observe any adverse effects of the digital counseling tool on cancer worry, state anxiety, subjective quality of life, or use of health care services. The content of the counseling was rated more positively by women in the IG compared with women in the CG.

Furthermore, our results show that both face-to-face and online counseling lead to similar outcomes for counselees, with no loss of quality. That is, the digital KNOW tool can be used by trained health professionals to provide counseling in person and online. This suggests that iKNOW could be an effective tool for disseminating counseling expertise and specialized counseling content within and across health care systems with varying degrees of coverage. A transfer of iKNOW to other countries that differ from the German setting is also possible because the information content is stored in a database that can be easily adapted to rapid changes in evidence. The established database also allows easy adaptation of the iKNOW tool to differences in languages, country-specific guidelines, and levels of content complexity.

### Risk understanding

It is likely that before genetic counseling, women do not view their risk in statistical terms.^23^ Genetic counseling with individually calculated risks aims to give an idea of numerical risk information. Our results indicate that supporting communication with a counseling tool that translates numerical risks into descriptive graphics improved risk understanding of breast and OC risks in that deviations of women’s estimates from actual risks in the IG seemed to be smaller. However, differences between the 2 groups showed only a trend after adjusting for baseline differences and accounting for multiplicity. The percentage of correct estimations of breast cancer risk was higher in the IG and remained significant after adjustment.

Although we had reached the original target sample size, some analyses were underpowered to reach significance because of missing values. In addition, the effects were slightly smaller than expected. Nevertheless, as all results are in the expected direction, we cautiously conclude that a dynamic graphical representation of risk has the potential to improve the accuracy of risk understanding.

Our results are consistent with and extend the exploratory analyses of the Australian tool iPrevent,^12^ which followed a similar approach but assessed risk perception with verbal labels. Because the term “risk perception” can cover different concepts, in our study, we distinguished between numerical risk understanding and verbal labels of subjective risk perception. Although numerical risk understanding involves expressing risk in quantitative terms, typically using numbers or statistical measures, verbal risk communication may use terms such as “low,” “medium,” or “high” to describe the perceived level of risk, without providing specific numerical values. With numerical risk communication, we provide a more precise and objective understanding of the risks communicated via the iKNOW tool.

We used a continuous variable to assess the difference between estimated and actual numerical risk information. For ease of interpretation, we have also dichotomized this variable into “correct” and “incorrect” estimates with a tolerance range of 5%. Each form of presentation has its advantages and disadvantages. For example, because the calculated risk for breast cancer is higher than that for OC and shows a greater variance, there are more underestimates and thus a smaller proportion of correct estimates. Conversely, the risk for OC, which in most women is in a lower range, is seldom underestimated because, with a 5% downward tolerance, there is little room for underestimation.

It is noteworthy that immediately after counseling, in both groups (IG and CG) the deviation from the actual risk decreased and the proportion of correct estimates increased, but these effects attenuated after 6 months (Supplemental Table 3). Furthermore, despite an increase of correct estimates after counseling, the percentage of overestimates remained high. Other studies that focused on numerical rather than verbal risk estimates of women with pathogenic germline variants in *BRCA1/2* also found that women generally tended to overestimate their risk, whereas a smaller proportion of women underestimated their risk.^23^^,^^24^ Both over- and underestimation carry risks. Although overestimation may be associated with more risk-reducing surgeries (eg, Haroun, et al,^25^ 2011), underestimation may delay preventive measures.

Apart from the statistical explanations mentioned above, misinterpretation of risk can also have psychological causes. First, the stressful situation may focus attention on the anxiety-provoking aspects. Several studies have shown that anxiety in genetic counseling is related to risk overestimation.^23^^,^^26^, ^27^, ^28^ Second, risk overestimation may be an expression of defensive pessimism (see also Gurmankin^24^), in which people anticipate the worst-case scenario but actively try to avoid it. Third, women may confuse 10-year risks and lifetime risks. Both risks are addressed in the counseling process, but the lifetime risk is particularly prominent in the media. Fourth, most women who come to the consultation had personal experiences with cancer in their families, often with close relatives, for instance, with siblings or parents. Such personal experiences do not necessarily correspond to the individual risk. The phenomenon that people tend to estimate the likelihood of an event based on its similarity to a particular prototype or stereotype has been well studied as the “representativeness heuristic.”^29^ Fifth, the sheer volume of information that is conveyed in the consultation may lead to mental overload and cause the meaning of the numbers to fade into the background. The fact that iKNOW had no effect on the high-level construct of cancer worry is in line with findings related to decision aids for counselees that are heterozygous for a pathogenic BRCA germline variant.^30^ As a consequence, interventions that target anxiety more directly are needed. Ultimately, dealing with anxiety will always remain one of the most important tasks of the physician and must be trained.^31^

### Strengths and limitations

The strengths of the study include the randomized design, which, combined with a standardized counseling situation, ensured high internal validity. In spite of difficult conditions during the COVID-19 pandemic, we achieved the recruitment target. The low dropout rate shows the high motivation of women with a pathogenic germline variant in BRCA.

Several limitations should be considered when interpreting the data: (1) complete blinding of the counselors was not possible because the intervention (counseling with vs without the tool) was obvious in each case. This bias was addressed by using a strict standardized study protocol in both IG and CG consistent with the standards of the GC-HBOC. (2) The intervention was tested against a standard condition in which the physicians already had a lot of experience with transparent risk communication. We suspect that the effects of the intervention would be greater for less experienced physicians. (3) The SARS-CoV-2 restrictions, which came into force in April 2020, led to a switch to video consultation and online questionnaires in 103 cases. However, a comparison between video consultation and face-to-face consultation showed no differences in the primary outcome (Supplemental Table 9). (4) The last follow-up took place after 6 months. Because women with a pathogenic variant of BRCA often take measures such as prophylactic surgery later in life, differences in mental health may also emerge later. Therefore, we suggest longer follow-up for future studies. (5) The power calculation resulted in a sample of *N* = 160 participants, which was achieved. However, missing values reduced the power to detect meaningful differences between IG and CG. In particular, counselees who did not provide an estimate were more often CG, more often diseased, and had a lower education. We also assume that women with poor risk understanding are more likely not to answer the question about a risk estimate.

### iKNOW improves process quality

iKNOW did not have an effect on patient-reported outcomes (PRO) such as anxiety or quality of life. Given the focus of digital technologies on improving efficiency, this sobering “nonresult” with regard to PROs draws attention to other parameters that measure process quality. In our view, this is where the greatest potential for digital applications is. Results from accompanying semistructured interviews and observations (see Schmid et al^32^) showed that the iKNOW counseling tool affected process quality for both doctors and their patients. For example, doctors reported that they were less likely to forget topics because of the standardized process. Patients, on the other hand, found it reassuring to know that they could review the content at home and thus focus more on the counseling session as it unfolds. We hope that improving procedures with digital technologies will create more time for doctor-patient interactions. This time could be deliberately used to better address patients’ concerns and fears. We also hope that as people become more comfortable using digital health apps, tools such as iKNOW will become more integrated into their daily lives, further improving health literacy.

### Conclusion

Our results show that a digital counseling tool that translates complex risk information into easy-to-understand formats may improve the understanding of individual cancer risk. In terms of PROs, digital tools such as our counseling tool are comparable to standard counseling. The evidence-based approach used to develop iKNOW can be seen as a paradigm for the development of digital innovations to support patient counseling and improve counseling quality. However, greater and maybe different efforts are required to further improve patient’s understanding of numerical risk information.

## Data Availability

The data that support the findings of this study are available on request from the corresponding author Friederike Kendel; email: friederike.kendel@charite.de. The data are not publicly available because of ethical restrictions.

## ORCID

Friederike Kendel: http://orcid.org/0000-0002-3726-0338

## Conflict of Interest

The authors declare no conflicts of interest.
