# Supplementary material for: iKNOW—Supporting the counseling of women with hereditary risk of breast and ovarian cancer with digital technologies: A randomized controlled trial
Source: Genet Med Open. 2024 Sep 6;2(Suppl 2):101892. doi: 10.1016/j.gimo.2024.101892 (PMC11658556; doi:10.1016/j.gimo.2024.101892)
Supplement: Supplementary Material [file mmc1.docx]

## Supplementary Material

**Supplementary Remark 1.** Formula for Box-Cox transformation of the outcome

We observed that the absolute deviation is empirically right-skewed. In order to better satisfy the assumptions of linear regression for adjusting for baseline values, we decided to apply a Box-Cox transformation to the outcome variables. This transformation ensures that the regression residuals are approximately normally distributed. The transformation is defined as follows:

$$Y_{i}^{\lambda}=\left\{ \begin{aligned} \frac{{Y_{i}}^{\lambda}-1}{\lambda} for \lambda\neq0 \\ \ln\left( Y_{i} \right) for \lambda=0. \end{aligned} \right.$$

Here, the Parameter $\lambda$ is determined using Maximum Likelihood estimation. For the ovarian cancer outcome, we found $\lambda=0.14$ and for the breast cancer outcome, we found$\lambda=0.34$.

**Supplementary Table 1.** Result tables for adjusted and unadjusted analysis for the absolute risk deviation and the dichotomized outcome at T3

| Absolute Risk Deviation |  | Ovarian cancer | Breast cancer |
| --- | --- | --- | --- |
| Unadjusted  (Mann-Whitney-U test) | **Relative treatment effect P(CG > IG)** | 0.60 | 0.57 |
|  | **95% CI** | [0.51, 0.70] | [0.48, 0.66] |
|  | **p-value** | 0.042*^a^* | 0.143 |
| Adjusted (Linear regression with baseline adjustment on Box-Cox-transformed outcome) | **Adjusted mean difference** | -0.64 | -0.74 |
|  | **95% CI** | [-1.3, 0.05] | [-1.7, 0.17] |
|  | **p-value** | 0.070 | 0.109 |

**Table 1a.** Absolute risk deviation

*^a^*p < 0.05

**Table 1b.** Dichotomized outcome

| Dichotomized outcome |  | Ovarian cancer | Breast cancer |
| --- | --- | --- | --- |
| Unadjusted (Chi-square test) | **OR** | 1.6 | 2.6 |
|  | **95% CI** | [0.84, 3.2] | [1.1, 5.8] |
|  | **p-value** | 0.204 | 0.032*^a^* |
| Adjusted (Logistic regression with baseline adjustment)) | **Adjusted OR** | 1.4 | 2.5 |
|  | **95% CI** | [0.66, 2.8] | [1.1, 5.8] |
|  | **p-value** | 0.407 | 0.027*^a^* |

*^a^*p < 0.05

Here, exemplarily, P(CG > IG) = 0.60 in case of ovarian cancer means absolute risk deviation values tend to be larger for the control group. More precisely, the estimated probability for a random absolute risk deviation from the control group to be larger than a random absolute risk deviation from the intervention group is 0.6. Regarding the dichotomized outcome, the OR = 1.6 means that the odds for a correct estimation (with a threshold of 5%) are 1.6 times higher for the intervention group.

**Supplementary Table 2.** Underestimations, overestimations, correct estimations and missing values of the primary outcome by time point and group. Note that estimates in this table differ from the primary analyses in the manuscript that do not include missing values.

We conducted an investigation into the frequency of underestimations, overestimations, correct estimations and missing values by time and group. In order to focus solely on participants without a subjective risk estimation, we excluded any missing values that were a result of a missing calculated risk at baseline. For ovarian cancer, this results in the inclusion of n = 47 observations. For breast cancer, we excluded n = 36 observations, resulting in a reduction in the overall sample size at each time point.

**Table 2a. Ovarian Cancer**

| Group |  | T0 | T1 | T2 | T3 |
| --- | --- | --- | --- | --- | --- |
| IG | **Underestimated, n (%)** | 4 (4.3) | 4 (4.3) | 3 (3.3) | 5 (5.4) |
|  | **Overestimated, n (%)** | 52 (56.5) | 28 (30.4) | 34 (37.0) | 28 (30.4) |
|  | **Correct Estimation, n (%)** | 33 (35.9) | 56 (60.9) | 45 (48.9) | 42 (45.7) |
|  | **Missing, n (%)** | 3 (3.3) | 4 (4.3) | 10 (10.9) | 17 (18.5) |
| CG | **Underestimated, n (%)** | 1 (1.3) | 2 (2.6) | 3 (3.8) | 2 (2.6) |
|  | **Overestimated, n (%)** | 60 (76.9) | 34 (43.6) | 38 (48.7) | 34 (43.6) |
|  | **Correct Estimation, n (%)** | 16 (20.5) | 39 (50.0) | 27 (34.6) | 28 (35.9) |
|  | **Missing, n (%)** | 1 (1.3) | 3 (3.8) | 10 (12.8) | 14 (17.9) |

T0: Baseline; T1: Immediately after intervention; T2: 4 weeks after intervention; T3: 6 months after intervention; IG: intervention groups; CG: control group

**Table 2b. Breast Cancer**

| Group |  | T0 | T1 | T2 | T3 |
| --- | --- | --- | --- | --- | --- |
| IG | **Underestimated, n (%)** | 28 (30.1) | 28 (30.1) | 28 (30.1) | 26 (28.0) |
|  | **Overestimated, n (%)** | 50 (53.8) | 30 (32.3) | 37 (39.8) | 26 (28.0) |
|  | **Correct Estimation, n (%)** | 13 (14.0) | 33 (35.5) | 21 (22.6) | 23 (24.7) |
|  | **Missing, n (%)** | 2 (2.2) | 2 (2.15) | 7 (7.5) | 18 (19.4) |
| CG | **Underestimated, n (%)** | 19 (21.6) | 26 (29.5) | 17 (19.3) | 21 (23.9) |
|  | **Overestimated, n (%)** | 59 (67.0) | 41 (46.6) | 42 (47.7) | 43 (48.9) |
|  | **Correct Estimation, n (%)** | 9 (10.2) | 18 (20.5) | 20 (22.7) | 11 (12.5) |
|  | **Missing, n (%)** | 1 (1.1) | 3 (3.4) | 9 (10.2) | 13 (14.8) |

T0: Baseline; T1: Immediately after intervention; T2: 4 weeks after intervention; T3: 6 months after intervention; IG: intervention groups; CG: control group

**Supplementary Table 3.** Comparison of the different time points within the groups

To examine the impact of counseling over time within the groups, we conducted pairwise comparisons between different time points using a nonparametric t-test, implemented using the R package nparcomp (Version 3.0). The results are presented below and show that the largest effect, observed in both treatment arms, is seen when comparing with the baseline. However, it is important to note that following this initial effect, the absolute risk deviation tends to increase again.

| Ovarian Cancer | | T0 vs T1 | T0 vs T2 | T0 vs T3 | T1 vs T2 | T1 vs T3 | T2 vs T3 |
| --- | --- | --- | --- | --- | --- | --- | --- |
| CG | **P(post > pre)** | 0.31 | 0.36 | 0.37 | 0.56 | 0.58 | 0.50 |
|  | **n** | 74 | 68 | 63 | 66 | 62 | 60 |
|  | **95% CI** | [0.24, 0.38] | [0.29, 0.43] | [0.30, 0.45] | [0.48, 0.64] | [0.50, 0.66] | [0.44, 0.56] |
|  | **p-value** | < 0.001*^a^* | < 0.001*^a^* | 0.002*^a^* | 0.140 | 0.046 | 0.965 |
| IG | **P(post > pre)** | 0.33 | 0.39 | 0.37 | 0.56 | 0.55 | 0.49 |
|  | **n** | 86 | 80 | 73 | 82 | 74 | 70 |
|  | **95% CI** | [0.27, 0.40] | [0.32, 0.46] | [0.29, 0.45] | [0.50, 0.62] | [0.48, 0.61] | [0.42, 0.55] |
|  | **p-value** | < 0.001*^a^* | 0.002*^a^* | 0.002*^a^* | 0.040*^a^* | 0.158 | 0.664 |
| ^a^p < 0. 05  T0: Baseline; T1: Immediately after intervention; T2: 4 weeks after intervention; T3: 6 months after intervention; IG: intervention groups; CG: control group | | | | | | | |

| Breast Cancer | | T0 vs T1 | T0 vs T2 | T0 vs T3 | T1 vs T2 | T1 vs T3 | T2 vs T3 |
| --- | --- | --- | --- | --- | --- | --- | --- |
| CG | **P(post > pre)** | 0.38 | 0.41 | 0.43 | 0.53 | 0.57 | 0.54 |
|  | **n** | 84 | 74 | 74 | 77 | 73 | 72 |
|  | **95% CI** | [0.31, 0.45] | [0.34, 0.48] | [0.36, 0.51] | [0.45, 0.60] | [0.48, 0.65] | [0.47, 0.60] |
|  | **p-value** | 0.001*^a^* | 0.011*^a^* | 0.089 | 0.452 | 0.114 | 0.269 |
| IG | **P(post > pre)** | 0.34 | 0.42 | 0.40 | 0.59 | 0.55 | 0.49 |
|  | **n** | 89 | 84 | 74 | 86 | 74 | 72 |
|  | **95% CI** | [0.27, 0.41] | [0.33, 0.50] | [0.31, 0.49] | [0.53, 0.65] | [0.47, 0.63] | [0.42, 0.56] |
|  | **p-value** | <0.001*^a^* | 0.055 | 0.031*^a^* | 0.005*^a^* | 0.220 | 0.709 |
| ^a^p < 0. 05  T0: Baseline; T1: Immediately after intervention; T2: 4 weeks after intervention; T3: 6 months after intervention; IG: intervention groups; CG: control group | | | | | | | |

**Supplementary Table 4.** Investigation of Missing Group at T3

We conducted an investigation into the demographic characteristics of a specific group of participants who had missing outcome values at T3. In order to focus solely on participants without a subjective risk estimation, we excluded any missing values that were a result of a missing calculated risk. This led to a reduction in the overall sample size.

|  | Ovarian Cancer Total | | | Breast Cancer Total | | |
| --- | --- | --- | --- | --- | --- | --- |
|  | **Missing**  **(*N* = 31)** | **Non-Missing**  **(*N* = 139)** | ***p*-Value** | **Missing**  **(*N* = 31)** | **Non-Missing**  **(*N* = 150)** | ***p*-Value** |
| Sociodemographic characteristics | |  |  |  |  |  |
| Age, yrs, *M (SD)* | 38.5 (11.1) | 37.3 (9.0) | 0.875*^2^* | 40.7 (12.9) | 39.5 (10.7) | 0.870*^2^* |
| With partner, *n* (%) | 22 (71.0) | 98 (71.5) | 1.000*^3^* | 20 (64.5) | 102 (68.9) | 0.751*^3^* |
| Higher education*^1^*, *n* (%) | 20 (64.5) | 109 (79.0) | 0.139*^3^* | 21 (67.7) | 112 (75.2) | 0.488*^3^* |
| Employed, *n* (%) | 15 (48.4) | 96 (69.1) | 0.048*^3^* | 15 (48.4) | 100 (66.7) | 0.085*^3^* |
| Biological children, *n* (%) | 18 (58.1) | 72 (52.9) | 0.751*^3^* | 18 (58.1) | 79 (54.1) | 0.839*^3^* |
| Type of the pathogenic variant | |  |  |  |  |  |
| *BRCA1*, *n* (%) | 19 (61.3) | 88 (63.3) | 0.996*^3^* | 19 (61.3) | 97 (64.7) | 0.880*^3^* |
| *BRCA2*, *n* (%) | 12 (38.7) | 51 (36.7) | 0.996*^3^* | 12 (38.7) | 53 (35.3) | 0.880*^3^* |
| Additional clinical variables |  |  |  |  |  |  |
| Diagnosis of cancer, *n* (%) | 16 (53.3) | 52 (37.4) | 0.159*^3^* | 16 (51.6) | 58 (39.2) | 0.282*^3^* |
| Breast cancer,  *n* (% of patients) | 15 (93.8) | 51 (98.1) | 0.960*^3^* | 15 (93.8) | 48 (82.8) | 0.486*^3^* |
| Ovarian cancer,  *n* (% of patients) | 0 (0.0) | 0 (0.0) | NA | 1 (6.3) | 10 (17.2) | 0.486*^3^* |
| Others,  *n* (% of patients) | 1 (6.3) | 1 (2.0) | 0.960*^3^* | 0 (0.0) | 0 (0.0) | NA |
| Missing, *n* | 1 | 0 | NA | 0 | 2 | NA |
| Familial cancer, *n* (%) | 28 (93.3) | 133 (95.7) | 0.940*^3^* | 28 (90.3) | 143 (95.3) | 0.497*^3^* |
| Missing, *n* | 1 | 0 | NA | 0 | 0 | NA |
| Prophylactic surgery, *n* (%) | 3 (10.0) | 20 (14.6) | 0.712*^3^* | 3 (9.7) | 20 (13.6) | 0.766*^3^* |
| Mastectomy,  *n* (% of patients) | 3 (100.0) | 19 (95.0) | 1.000*^3^* | 0 (0.0) | 8 (40.0) | 0.480*^3^* |
| Unilateral,  *n* (% of patients) | 0 (0.0) | 4 (21.1) | 0.942*^3^* | 0 (NA) | 6 (75.0) | NA |
| Bilateral,  *n* (% of patients) | 3 (100.0) | 15 (78.9) | 0.942^3^ | 0 (NA) | 2 (25.0) | NA |
| Oophorectomy,  *n* (% of patients) | 0 (0.0) | 1 (5.0) | 1.000*^3^* | 3 (100.0) | 15 (75.0) | 0.819*^3^* |
| Salpingectomy,  *n* (% of patients) | 0 (0.0) | 1 (5.0) | 1.000*^3^* | 0 (0.0) | 5 (25.0) | 0.819*^3^* |
| Missing, *n* | 1 | 2 | NA | 0 | 3 | NA |

^1^defined as German “Abitur” (i.e., final exam after 12 to 13 grades of schooling) ^2^Mann-Whitney U test; ^3^Chi-square test;

**Supplementary Table 5.** Descriptive statistics and linear mixed model of risk perception

|  |  | T0 | T1 | T2 | T3 |
| --- | --- | --- | --- | --- | --- |
| IG | **Mean** | 6.1 | 5.2 | 5.5 | 5.0 |
|  | **Median** | 6.0 | 5.0 | 5.0 | 5.0 |
|  | **25% Percentile** | 4.0 | 3.0 | 3.0 | 2.5 |
|  | **75% Percentile** | 8.0 | 7.3 | 8.0 | 8.0 |
|  | **SD** | 2.7 | 2.8 | 2.7 | 3.0 |
|  | **n** | 105 | 102 | 95 | 85 |
| CG | **Mean** | 6.1 | 5.1 | 5.3 | 5.0 |
|  | **Median** | 7.0 | 5.0 | 5.0 | 5.0 |
|  | **25% Percentile** | 5.0 | 3.0 | 3.0 | 3.0 |
|  | **75% Percentile** | 8.0 | 7.8 | 7.0 | 7.0 |
|  | **SD** | 2.3 | 2.6 | 2.5 | 2.5 |
|  | **n** | 112 | 108 | 100 | 94 |
|  | **p-value*^1^*** | 0.931 | 0.913 | 0.428 | 0.987 |

T0: Baseline; T1: Immediately after intervention; T2: 4 weeks after intervention; T3: 6 months after intervention; IG: intervention groups; CG: control group **^1^**Mann-Whitney-U Test

|  | Intercept*^1^* | 95%CI | | Slope*^2^* | 95%CI | | p-value*^3^* |
| --- | --- | --- | --- | --- | --- | --- | --- |
| IG | 5.3 | 5.0 | 5.6 | -0.05 | -0.13 | 0.04 | 0.277 |
| CG | 5.1 | 4.8 | 5.5 | -0.04 | -0.12 | 0.04 | 0.347 |
| p-value*^4^* | 0.454 |  |  | 0.885 |  |  |  |

IG: Intervention group, CG: Control group

Linear mixed model with baseline, time (in months) since intervention and control group (IG/CG) as covariates and a fixed and random intercept.

^1^Intercept: mean risk perception after 2,3 months after intervention.

^2^Slope: Change in subjective risk perception per months.
^3^Test of null hypothesis Slope = 0.

^4^Test of comparison between CG and IG.

**Supplementary Table 6.** Descriptive statistics and linear mixed model of illness anxiety

|  |  | T0 | T1 | T2 | T3 |
| --- | --- | --- | --- | --- | --- |
| IG | **Mean** | 11.1 | 10.6 | 10.0 | 9.8 |
|  | **Median** | 11.0 | 11.0 | 10.0 | 10.0 |
|  | **25% Percentile** | 10.0 | 9.0 | 7.0 | 7.0 |
|  | **75% Percentile** | 14.0 | 13.0 | 13.0 | 12.0 |
|  | **SD** | 3.1 | 3.0 | 3.2 | 3.3 |
|  | **n** | 104 | 67 | 94 | 83 |
| CG | **Mean** | 10.9 | 10.5 | 10.4 | 9.6 |
|  | **Median** | 11.0 | 11.0 | 11.0 | 10.0 |
|  | **25% Percentile** | 8.8 | 8.0 | 7.3 | 6.5 |
|  | **75% Percentile** | 14.0 | 13.0 | 13.0 | 12.0 |
|  | **SD** | 3.2 | 3.1 | 3.3 | 3.4 |
|  | **n** | 110 | 72 | 100 | 93 |
|  | **p-value*^1^*** | 0.666 | 0.906 | 0.460 | 0.668 |

T0: Baseline; T1: Immediately after intervention; T2: 4 weeks after intervention; T3: 6 months after intervention; IG: intervention groups; CG: control group **^1^**Mann-Whitney-U Test

|  | Intercept*^1^* | 95%CI | | Slope*^2^* | 95%CI | | p-value*^3^* |
| --- | --- | --- | --- | --- | --- | --- | --- |
| IG | 10.1 | 9.7 | 10.4 | -0.10 | -0.20 | 0.00 | 0.051 |
| CG | 10.2 | 9.9 | 10.6 | -0.12 | -0.22 | -0.03 | 0.011 |
| p-value*^4^* | 0.580 |  |  | 0.725 |  |  |  |

IG: Intervention group, CG: Control group

Linear mixed model with baseline, time (in months) since intervention and group (IG/CG) as covariates and a fixed and random intercept.

^1^Intercept: mean risk perception after 2,3 months after intervention.

^2^Slope: Change in subjective risk perception per months.

^3^Test of null hypothesis Slope = 0.

^4^Test of comparison between CG and IG

**Supplementary Table 7.** Descriptive statistics and linear mixed model of general anxiety

|  |  | T0 | T1 | T2 | T3 |
| --- | --- | --- | --- | --- | --- |
| IG | **Mean** | 10.3 | 9.1 | 9.6 | 9.8 |
|  | **Median** | 10.0 | 8.0 | 8.0 | 9.0 |
|  | **25% Percentile** | 7.0 | 6.0 | 6.0 | 7.0 |
|  | **75% Percentile** | 12.0 | 11.0 | 12.5 | 11.0 |
|  | **SD** | 4.0 | 3.7 | 4.1 | 3.9 |
|  | **N** | 103 | 99 | 97 | 84 |
| CG | **Mean** | 10.3 | 8.9 | 9.7 | 9.5 |
|  | **Median** | 10.0 | 8.0 | 9.0 | 10.0 |
|  | **25% Percentile** | 8.0 | 6.0 | 7.0 | 7.0 |
|  | **75% Percentile** | 12.0 | 10.0 | 12.3 | 11.0 |
|  | **SD** | 3.5 | 3.2 | 3.6 | 3.2 |
|  | **N** | 109 | 107 | 98 | 92 |
|  | **p-value*^1^***^1^ | 0.705 | 0.936 | 0.500 | 0.869 |

T0: Baseline; T1: Immediately after intervention; T2: 4 weeks after intervention; T3: 6 months after intervention; IG: intervention groups; CG: control group ^1^Mann-Whitney-U Test

|  | Intercept*^1^* | 95%CI | | Slope*^2^* | 95%CI | | p-value*^3^* |
| --- | --- | --- | --- | --- | --- | --- | --- |
| IG | 9.5 | 9.1 | 9.9 | 0.07 | -0.03 | 0.17 | 0.151 |
| CG | 9.4 | 9.0 | 9.7 | 0.09 | 0.00 | 0.19 | 0.061 |
| p-value*^4^* | 0.597 |  |  | 0.791 |  |  |  |

IG: Intervention group, CG: Control group

Linear mixed model with baseline, time (in months) since intervention and group (IG/CG) as covariates and a fixed and random intercept.

^1^Intercept: mean risk perception after 2,3 months after intervention.

^2^Slope: Change in subjective risk perception per months.

^3^Test of null hypothesis Slope = 0.

^4^Test of comparison between CG and IG

**Supplementary Table 8.** Descriptive statistics of quality of life

|  | T0 | | T2 | | T3 | |
| --- | --- | --- | --- | --- | --- | --- |
|  | **IG** | **CG** | **IG** | **CG** | **IG** | **CG** |
| Excellent | 4.5% | 5.3% | 5.0% | 6.8% | 8.2% | 8.3% |
| Very good | 29.1% | 24.6% | 26.7% | 25.2% | 24.7% | 17.7% |
| Good | 39.1% | 37.7% | 41.6% | 41.7% | 52.9% | 56.3% |
| Rather bad | 24.5% | 27.2% | 25.7% | 22.3% | 10.6% | 14.6% |
| Bad | 2.7% | 5.3% | 1.0% | 3.9% | 3.5% | 3.1% |
| n | 110 | 114 | 101 | 103 | 85 | 96 |
| p-value*^1^*^1^ | 0.390 | | 0.989 | | 0.445 | |

T0: Baseline; T1: Immediately after intervention; T2: 4 weeks after intervention; T3: 6 months after intervention; IG: intervention groups; CG: control group

^1^Chi-Square test

**Supplementary Table 9.** Comparison of the primary outcome between video consultation and face-to-face consultation for T0 – T3

|  |  | n | % missing | Mean | SD | Median | 25% Percentile | 75% Percentile |
| --- | --- | --- | --- | --- | --- | --- | --- | --- |
| T0 |  |  |  |  |  |  |  |  |
|  | **Video** | 86 | 13.1 | 27.4 | 18.5 | 24.0 | 11.9 | 42.4 |
|  | **Face-to-Face** | 92 | 20.7 | 23.6 | 19.6 | 18.2 | 5.9 | 37.6 |
|  | **p-value***^1^* |  | 0.078 |  |  |  |  |  |
| T1 |  |  |  |  |  |  |  |  |
|  | **Video** | 83 | 16.2 | 17.8 | 18.2 | 10.5 | 4.3 | 28.1 |
|  | **Face-to-Face** | 93 | 19.8 | 16.4 | 15.1 | 13.1 | 4.2 | 26.5 |
|  | **p-value***^1^* |  | 0.957 |  |  |  |  |  |
| T2 |  |  |  |  |  |  |  |  |
|  | **Video** | 78 | 21.2 | 22.2 | 20.4 | 16.4 | 4.4 | 38.2 |
|  | **Face-to-Face** | 87 | 25.0 | 19.4 | 16.7 | 16.0 | 6.0 | 27.3 |
|  | **p-value***^1^* |  | 0.760 |  |  |  |  |  |
| T3 |  |  |  |  |  |  |  |  |
|  | **Video** | 78 | 21.2 | 21.3 | 19.6 | 14.3 | 5.8 | 32.7 |
|  | **Face-to-Face** | 72 | 37.9 | 21.0 | 17.9 | 16.1 | 5.5 | 29.9 |
|  | **p-value***^1^* |  | 0.765 |  |  |  |  |  |

^1^t-test
